# Flexible Wearable Sensors in Medical Monitoring

**DOI:** 10.3390/bios12121069

**Published:** 2022-11-23

**Authors:** Yingying Yuan, Bo Liu, Hui Li, Mo Li, Yingqiu Song, Runze Wang, Tianlu Wang, Hangyu Zhang

**Affiliations:** 1 Liaoning Key Lab of Integrated Circuit and Biomedical Electronic System, School of Biomedical Engineering, Dalian University of Technology, Dalian 116024, China; 2 Faculty of Medicine, Dalian University of Technology, Dalian 116024, China; 3 Department of Nursing, Cancer Hospital of Dalian University of Technology (Liaoning Cancer Hospital & Institute), Shenyang 110042, China; 4 Department of Radiotherapy, Cancer Hospital of Dalian University of Technology (Liaoning Cancer Hospital & Institute), Shenyang 110042, China; 5 School of Clinical Medicine, Chengdu Medical College, Chengdu 610500, China

**Keywords:** flexible wearable sensors, physiological signal monitoring, flexible substrates, printing technology, self-power

## Abstract

The popularity of health concepts and the wave of digitalization have driven the innovation of sensors in the medical field. Such continual development has made sensors progress in the direction of safety, flexibility, and intelligence for continuous monitoring of vital signs, which holds considerable promise for changing the way humans live and even treat diseases. To this end, flexible wearable devices with high performance, such as high sensitivity, high stability, and excellent biodegradability, have attracted strong interest from scientists. Herein, a review of flexible wearable sensors for temperature, heart rate, human motion, respiratory rate, glucose, and pH is highlighted. In addition, engineering issues are also presented, focusing on material selection, sensor fabrication, and power supply. Finally, potential challenges facing current technology and future directions of wearable sensors are also discussed.

## 1. Introduction

Since ancient times, people have struggled with diseases, so continuous innovation of medical equipment has always been the greatest goal of people’s struggle. However, many efficient medical facilities are primarily concentrated in hospitals, which makes health care time-consuming and labor-intensive, and the high cost of maintaining and purchasing these facilities can also create barriers for patients, especially in developing countries [1]. Accordingly, scientists are motivated to develop emerging technologies to implement healthcare services in a convenient and low-cost way, free patients from tedious hospital diagnoses, and meet the concept of personalized medicine spreads [2]. In the context, wearable medical devices for health monitoring emerge as the times require, monitoring vital signs for the wearer, especially heart rate [3], body temperature [4], and blood pressure [5,6].

**Figure 1:**
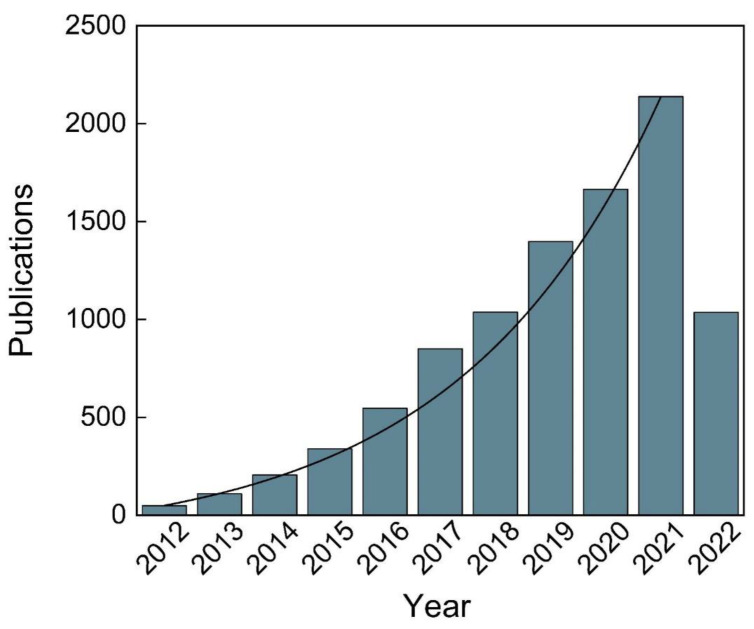
The number of publications on flexible wearable sensors in 2012–2022 (under the keyword of “flexible wearable sensors’’ in the Web of Science database).

The development and integration of materials science, sensing techniques, wireless technologies, and the internet of things have greatly promoted the advancement of wearable devices [7,8]. Wearable devices have become a new way to maintain a healthy life [1,9]. According to the data from the International Data Corporation (IDC) [10], global shipments of wearable devices have gradually increased. In the past two years, it has shown explosive growth. In 2020, despite being affected by the epidemic, its shipments still increased by 32% to 444.7 million yuan. It can be seen that wearable devices are still hot, and the market demand is also increasing [10]. Wearable devices for health monitoring are usually made into miniaturized rigid circuit boards and block power supplies placed on various parts of the human body, especially the wrist, to monitor body data in real-time [11]. However, these sensors lack sufficient flexibility and adaptability. Thus, the properties related to long-term wear comfort, including biocompatibility, skin compactness, safety, and durability, remain important considerations [12,13,14]. For the above purposes, research focusing on “flexible wearable sensors” is increasing. According to the Web of Science, the total number of publications on flexible wearable sensors has been increasing gradually in recent years, as depicted in Figure 1, showing a high interest in flexible wearable sensors.

**Figure 2:**
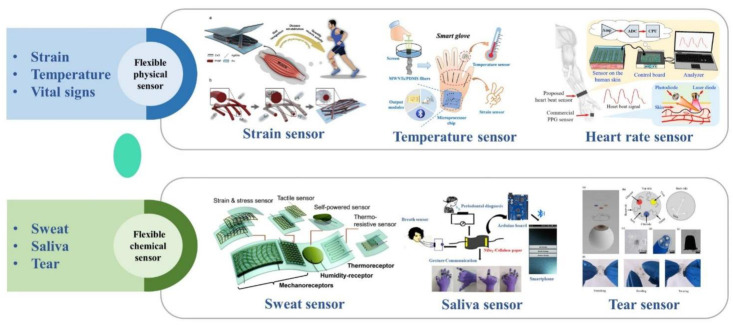
Examples of flexible physical sensors, including strain sensors, reprinted with permission from ref. [15]. Copyright 2020, Elsevier. Temperature sensor, reprinted with permission from ref. [16]. Copyright 2020, American Chemical Society. Heart rate sensor, reprinted with permission from ref. [17]. Copyright 2017, Elsevier. Flexible chemical sensors, including sweat sensor, reprinted with permission from ref. [18]. Copyright 2019, Royal Society of Chemistry. Saliva sensor, reprinted with permission from ref. [19]. Copyright 2019, American Chemical Society. Tear sensor, reprinted with permission from ref. [20]. Copyright 2020, SpringerLink.

Due to the complexity of the human body, various physiological signals need to be monitored in the medical field, so various sensors have been widely used. Flexible sensors for monitoring physical body signals, including strain, temperature, and vital signs, dominated the market in the early days. With increasing medical demand, chemical sensors that can reflect the physiological parameters of the human body have gradually become the focus in recent years. In order to better review the latest progress of flexible wearable sensors reported in the past few years, this paper introduces the application of sensors in the field of medical monitoring from the aspects of flexible materials, fabrication technology, and power supply methods according to the difference of monitoring signals. Figure 2 shows the evolution of flexible wearable sensors.

**Figure 3:**
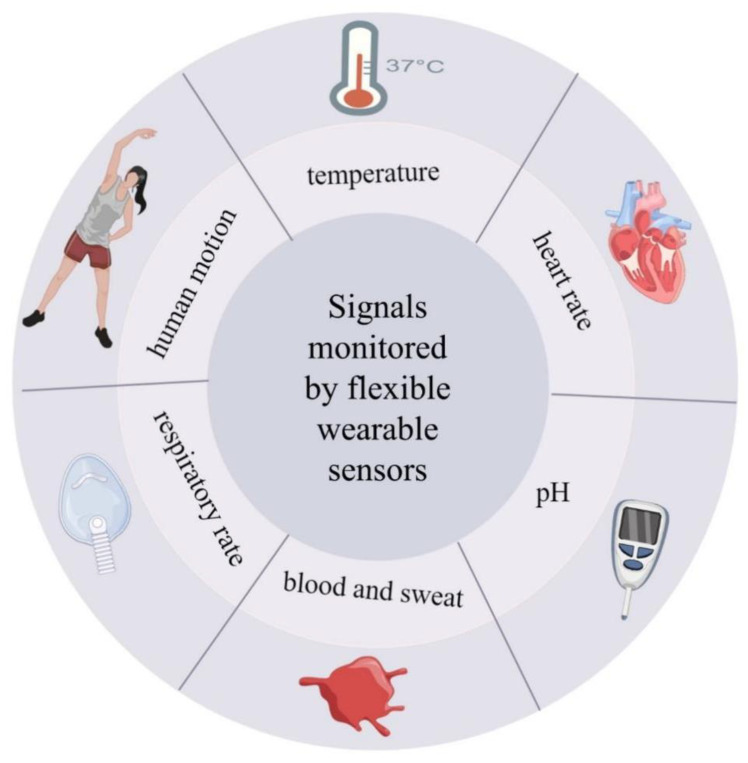
A visual summary of biological signals.

In the following sections, Section 2 briefly summarizes the commonly used flexible sensors in recent years for monitoring biological signals such as body temperature, respiration, heart rate, blood, exercise, and pH (Figure 3). General fabrication processes that allow flexibility are also reviewed in Section 4, following the application of flexible materials in Section 3. A scheme for the new power supply was proposed as an auxiliary part. Finally, this review concludes with the progress made in this field and the challenges that lie ahead.

## 2. Application of Flexible Wearable Sensors for Health-Monitoring

Impelled by the increasing demand for the elderly and various patients, especially those with chronic diseases, a variety of sensors have been developed in recent years to meet the need for continuous medical monitoring. For the needs of continuous medical detection, highly flexible, stretchable, safe, and reliable wearable sensors have gradually become a hot topic. Wearable flexible sensors can detect and monitor biological signals, such as pulse rate, respiration rate, temperature, exercise, and blood pressure, for timely diagnosis of disease [21,22,23].

A series of recent reports have shown that various configurations of flexible sensors, including piezoresistive sensors, piezo-electromechanical devices, capacitive sensors, and transistor-transistor-based devices, have demonstrated high sensitivity capable of monitoring human physiological signals [24,25,25,26,27,28,29]. Roughly, the indicators detectable in medical monitoring are classified into two categories: (1) biophysical signals, including body temperature, heart rate, pulse, and body movement; (2) biochemical signals, such as blood, pH, and biomolecules [30,31]. Monitoring heart rate, temperature, and human movement play a vital role in assessing health [32]. A summary of flexible sensors for vital sign monitoring is shown in Table 1.

### 2.1. Temperature

Body temperature is one of the critical indicators of daily health status and clinical diagnosis and treatment, providing insight into the physiological state of the human body. For example, the plantar temperature rise has proven to be a measurable indicator of diabetic foot ulceration [33,34]. The normal range of body temperature regulation is 36.5–37.1 °C [35], and abnormal body temperature often indicates a threat to the patient’s health. Maintaining body temperature within a healthy range is critical for blood circulation, the enzyme activity of the immune system, and physiological metabolism [36,37]. On the one hand, several diseases are characterized by irregular changes in body temperature [38] (physical activity and ambient temperature only cause slight changes in body temperature [39]). On the other hand, doctors can also accurately analyze the efficiency of treatment based on body temperature [40]. This indicator plays an integral role in medical health monitoring [41].

Changes in human body temperature are limited, so it is necessary to design flexible temperature sensors with high sensitivity and high accuracy to monitor the subtle changes in human body temperature. The traditional methods of measuring temperature include sticking temperature sensors and infrared digital cameras [42], but none of them can provide the function of continuous monitoring and cannot meet clinical needs. Therefore, many predecessors have devoted themselves to the development of new body temperature sensors. A flexible and stretchable temperature sensor array composed of single-walled carbon nanotubes was proposed, which can maintain stable mechanical properties under the external stain [43]. The flexible substrate of the sensor was polyaniline nanofiber, which can closely fit the skin. It adopted the embedded electrical connection method, with a precise response time of 1.8 s and high resistance sensitivity.

**Figure 4:**
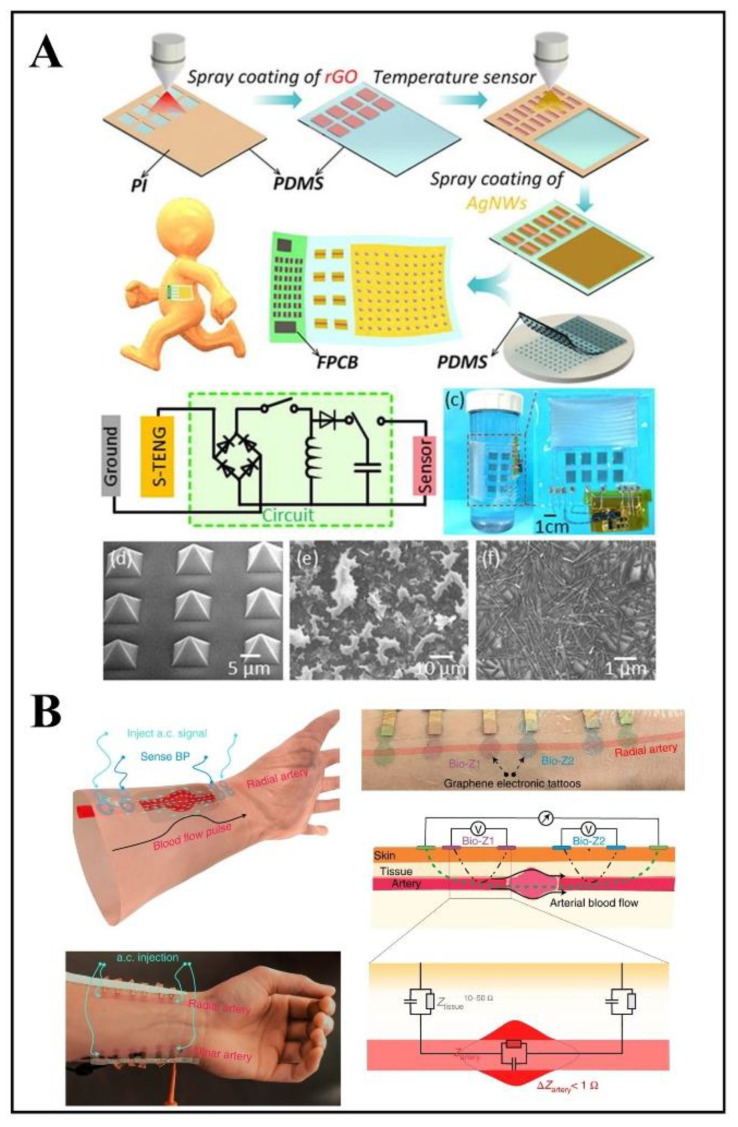
Examples of wearable flexible sensors for temperature and blood pressure monitoring. (**A**) A flexible and transparent smart patch with self-powered for temperature sensing, reprinted with permission from ref. [44]. Copyright 2020, Journal of Applied Physics. (**B**) A flexible blood pressure monitoring device, illustration of Z-BP measurement modality, reprinted with permission from ref. [55]. Copyright 2022, Nature.

Many types of flexible temperature sensors have been proposed, but the key to real-world application is that flexible wearable devices must have the ability to supply energy continuously. Recently, a new type of flexible temperature sensor system has been reported, which realized the integrated design of energy supply and temperature sensing(Figure 4A) [44]. Temperature arrays and triboelectric nanogenerators were integrated with power management circuits on the flexible polydimethylsiloxane (PDMS) substrate to measure subtle temperature changes. Among them, the triboelectric nanogenerator was used to generate energy, and the power management circuit was used to solve the problem of the large impedance of the triboelectric nanogenerator to achieve an effective power supply. Temperature sensors can not only monitor changes in human body temperature but also monitor changes in external ambient temperature [45]. An ultra-thin, lightweight, multi-function sensor was proposed for continuously monitoring light intensity and ambient temperature to avoid excessive or low ambient temperature hindering the healing of certain diseases and wounds [46]. The photodetectors and temperature sensors were integrated on the polyetherimide (PEI) soft substrate by lithography and etching techniques. The overall thickness of the sensor is within 20 μm, and is compliant with any part of the body without discomfort to wearers. All of the above innovations in temperature sensors for various applications demonstrate the extreme interest of the entire community of researchers.

### 2.2. Heart Rate and Pulse

Continuous monitoring of heart rate and pulse is essential for the prevention and diagnosis of cardiovascular disease. The main function of the human heart is to pump blood to the tissues and organs of the whole body while recycling venous blood and completing the exchange of substances [8]. Heart rate is the frequency of the cardiac cycle. In clinical practice, heart rate and pulse are the two most basic indicators of the heart [4,47]. Cardiovascular disease is one of the leading causes of human death, with cardiovascular disease (CVD) accounting for more than 17 million deaths annually, accounting for 31% of global deaths [48], and this number is expected to increase to 23.6 million by 2030 [49]. Despite high mortality, 90% of CVD can be prevented with early detection [50]. However, heart rate and pulse provide necessary information for timely prevention and treatment of cardiovascular disease [51].

At very early stages, cardiovascular disease is asymptomatic but causes arterial pulse pathology, affects arterial blood pressure, and causes changes in the pulse waveform at the wrist [52]. At present, traditional cuff sphygmomanometers are still used in the clinic to monitor blood pressure by measuring systolic and diastolic blood pressure [53,54], which is an indirect monitoring method and cannot meet the need for continuous monitoring. A flexible blood pressure monitoring device that easily sticks graphene tattoo sheets on the body has been designed with the characteristics of being ultralight and thin (Figure 4B) [55]. The tattoo was placed on an artery in the wrist, and the electrical current was injected into the skin to analyze the body’s response (bioimpedance). The correlation between bioimpedance and blood pressure changes was analyzed using machine learning models to create a database for continuous blood pressure monitoring. Another study on the flexible blood pressure sensor utilized piezoelectric composite ultrasonic technology different from previous blood pressure monitoring methods [56]. In this study, the flexible properties of the sensors were formed by integrating PDMS and conductive silver nanowire by a dice-and-fill technique.

Often, the activity of the heart can be represented by the electrocardiogram (ECG) signal, which provides general information about the cardiovascular system. Since the ECG is periodic, the heart rate can be obtained from the R wave-to-R wave (RR) interval of the ECG signal [8]. Traditional monitoring methods generally use gel-assisted Ag/AgCl, but this method is still relatively cumbersome to use [57]. The skin-contactable ECG sensor allows users to easily identify their cardiac condition and diagnose critical cardiac problems early, such as cardiomyopathy and arrhythmia, and high blood pressure [58,59]. The ECG electrodes are the key to monitoring ECG signals in flexible wearable devices. Traditional monitoring electrodes generally use gel-assisted Ag/AgCl electrodes, but there are still some troubles in using this method, such as causing skin problems [57,60]. With further research, flexible dry electrodes have gradually attracted extensive attention of researchers due to their soft and non-invasive characteristics. A flexible and wearable electrode was reported for ECG signal monitoring [61]. Through plasma sputtering technology, silver with excellent electrical conductivity was made on the substrate made of cowhide as a flexible material. In this work, the researchers performed ECG tests on six subjects and demonstrated that the monitored ECG signal quality was comparable to that of conventional Ag/AgCl electrodes. It is worth mentioning that this kind of cowhide material is easier to make clothes with integrated flexible sensors, and it is more easily accepted by people.

Plethysmography and ultrasound are commonly used methods for monitoring pulse and heart rate [62]. However, the bulky monitoring equipment and the accuracy of long-term monitoring prevent these two methods from becoming the best choice for wearable pulse sensors [63,64]. Recently, a flexible strain sensor for real-time pulse monitoring has been proposed, which can be easily attached to the skin [65]. The sensing element of this patch-type sensor adopted polyaniline with high sensitivity and excellent flexibility. Through the pressure changes caused by blood flow, the signal characteristics monitored by the pulse sensor were analyzed for clinical analysis. With more in-depth research, a flexible pressure sensor that can monitor three pulse positions simultaneously was designed [66]. This design combined traditional Chinese pulse theory with modern sensor technology to achieve innovation. Ionogel-based pressure sensor arrays were fabricated on polyethylene terephthalate (PET) flexible substrates to convert pressure changes brought about by arterial flow into resistance changes. The special feature of this research is that three-dimensional pulse mapping can be formed. The key information, including the strength and waveform of the pulse, is displayed on maps, perfectly simulating the sensation of a doctor touching the pulse on the skin. Another successful example of flexible sensors for monitoring pulse rate is based on poly(vinylidenefluoride-co-trifluoroethylene) (PVDF-TrFE) [67]. The device integrated analog amplification circuitry and detector elements on the flexible material that was capable of detecting weak pressures (10 kPa) and amplified electrical signals by a factor of 10. Therefore, the performance of the sensor is excellent for monitoring the pulse of the human body.

### 2.3. Human Motion

Correct exercise and fitness, medical rehabilitation training, and daily healthy life are inseparable from accurate and timely monitoring of human body movement signals. In daily life, detecting physical activity and exercise habits can provide useful information for fitness and maintaining correct posture. In the field of medicine, continuous monitoring and regular analysis of body movements will help doctors timely detect abnormalities in the patient’s body, thereby improving the efficiency of treatment. For example, abnormal posture and tremors in the hands are symptoms of some deadly diseases such as diabetes [68], Alzheimer’s [69], and Parkinson’s [70,71]. Wearable medical devices are essential in detecting sudden tremors and abnormal movements. Therefore, designing continuous monitoring sensors will help detect symptoms in time and treat them accurately and efficiently [72]. The normal movement of hands and limbs can ensure a high quality of life and efficient work, among which gestures are also vital and often serve as a range of signs to transmit information [73]. Given that the feet of the body bear most of the pressure of the body, especially for manual workers or athletes, monitoring the pressure changes on the soles of the feet can effectively prevent injuries [74,75,76].

To date, many efforts and achievements have been reported on flexible sensors for continuous monitoring of human motion. Wearable motion detection is mostly based on strain sensors, whereby the change in the base resistance is correlated with motion-related activities [77]. Strain sensors were developed using metal embedded into an elastomer and placed onto the hip, knee, and ankle joints to monitor their bending angles. A flexible strain sensor integrating sensing elements into textiles was proposed, which can effectively monitor the movement of the knee joint [78]. This study analyzed and guided sensor design based on the biomechanics of knee motion deformation. The sensing elements that monitor movement were encapsulated to avoid errors in measurement results. In addition, another special feature of this work was that they designed the sensor to be personalized, designed in different sizes and shapes to meet the comfort needs of different wearers. Long-term use of metals as electrodes may cause skin problems. A human motion detection device was developed using triboelectric nanogenerator (TENG) yarn, which has good skin compatibility and can be washed [79]. The device monitored body movement by attaching highly flexible and stretchable TENG fabric to different parts of the body, such as arms and knees. The TENG fabric was designed as a five-layer structure, of which the innermost layer was a coil spring. Since both ends of the coil spring are hook-shaped, they can be connected to each other to form an 11 × 11 array sensing fabric for multi-channel sensing. Surface-parallel TENG fabricated polytetrafluoroethylene yarns for large-scale energy harvesting. These works provide strong support for continuous monitoring of body movement, which should be widely used in the field of medical monitoring in the future. Recently, a magnetic tactile sensor was developed that distinguishes stretching and bending stimuli accordingly by opposite electrical signals [80]. The sensor used PDMS as the flexible substrate and mask-patterning and spin-coating technology to design a sandwich structure thin film sensor. The sandwich structure sensor attached to the surface of the skin was converted into the electrical signal output by feeling the pull force when the joint was bent. Since the sandwich structure sensor contained magnetic nanoparticles and the ability of self-sensing, it can be used as a non-contact magnetic sensor to effectively prevent the spread of bacteria in medical environments.

**Figure 5:**
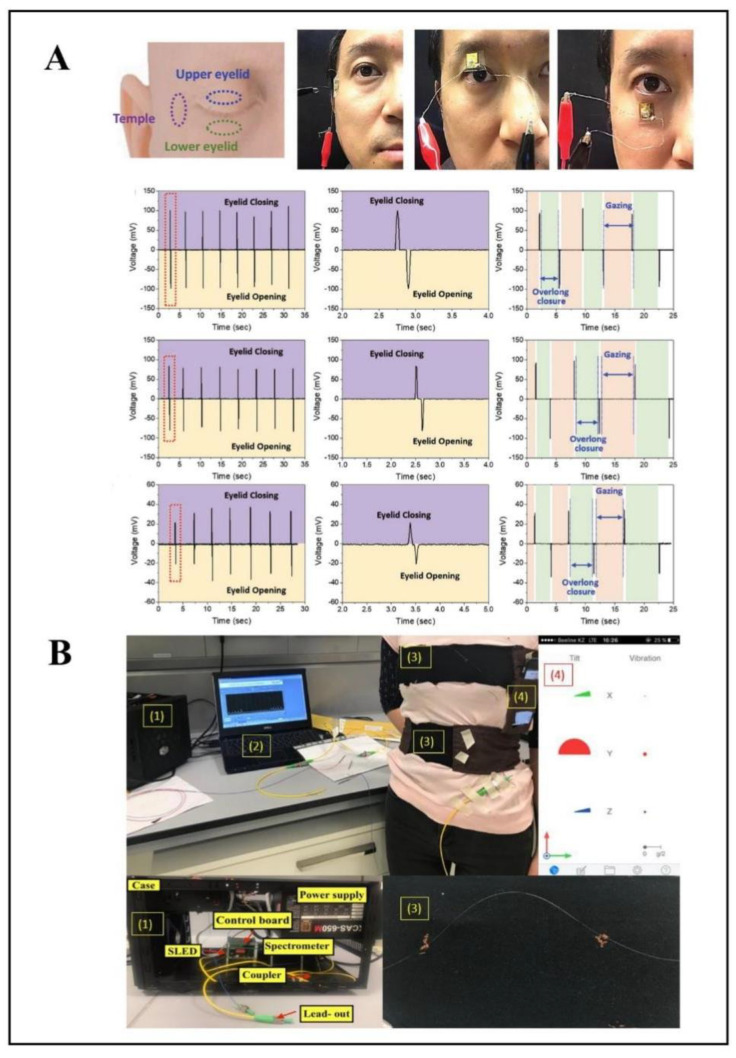
Examples of wearable flexible sensors for human motion and respiratory monitoring. (**A**) A noninvasive, and skin−attachable sensor using flexible piezoelectric thin film for monitoring the movement of the eye−blink, reprinted with permission from ref. [81]. Copyright 2021, Wiley. (**B**) A wearable system for respiratory monitoring with fiber-optic based smart textiles, (1) I-MON interrogator, (2) evaluation software, (3) belts equipped with an array of 5 FBGs, (4) VibSensor application. Reprinted with permission from ref. [89]. Copyright 2020, MDPI.

In addition to monitoring knee and wrist movements, subtle movements also need attention. For example, heavy use of various electronic devices and excessive workloads can lead to overuse of the eyes. Therefore, continuous monitoring of eye movement can prevent the occurrence of eye diseases in a timely manner and can also give timely prompts when the eyes are overloaded. A piezoelectric sensor that indicated eye fatigue by monitoring changes brought about by blinking was reported (Figure 5A) [81]. Unlike previous sensors directly attached to the eyelid, this work attached gallium nitride encapsulated in PDMS to the temple area on the face. When the sensor attached to the face changes due to sight or eye movement, it caused changes in the degree of curvature of the sensor, resulting in changes in the piezoelectric electrodes. The design offers promise for continuous monitoring of eye movement due to its small size, non-invasiveness, and high sensitivity.

### 2.4. Respiratory Rate

Monitoring respiratory rate has always been an important indicator of concern for doctors, patients, and those who care about their own physical health. With the outbreak of COVID-19 in recent years, people are paying more attention to monitoring respiratory rates. Therefore, the respiratory rate should be given more attention as a key aspect of vital signs because an abnormal respiratory rate is often a symptom of many diseases, such as sleep apnea, asthma, chronic obstructive pulmonary disease, anemia, nasal and sinus blockage, cough, and mild fever [82,83].

The commonly used respiratory rate monitoring technology is mainly the chest impedance method. In impedance plethysmography, electrodes are placed onto the body, and the change in impedance between them reflects the change in lung volume during inhalation and exhalation [84]. However, long-term attachment to the skin can cause discomfort. Due to their simplicity, stretchability, small size, and immunity to electromagnetic interference, fiber Bragg grating (FBG) sensors are widely used in respiratory rate monitoring applications [85,86]. When the FBG sensor monitors the respiratory rate, it is usually based on two methods. First, the FBG sensor records the respiratory rate by monitoring the change in the chest volume during the breathing process. Another way is to monitor respiration rate by differences in temperature and humidity during respiration [87]. A sensor designed to monitor the breathing rate of workers working under high pressure was proposed, which equipped the FBG to the clothing to form a smart clothing system [88]. The polyimide (PI) was used as a fiber coating, and then the FBG was encapsulated in a flexible matrix, which can improve the performance of the sensor and accommodate people of different body sizes. Based on the same principle, converting physical changes were brought about by breathing into the wavelength shift, and another study integrated five FBG sensors into the elastic band of textiles for respiratory rate monitoring (Figure 5B) [89].

### 2.5. Glucose

**Figure 6:**
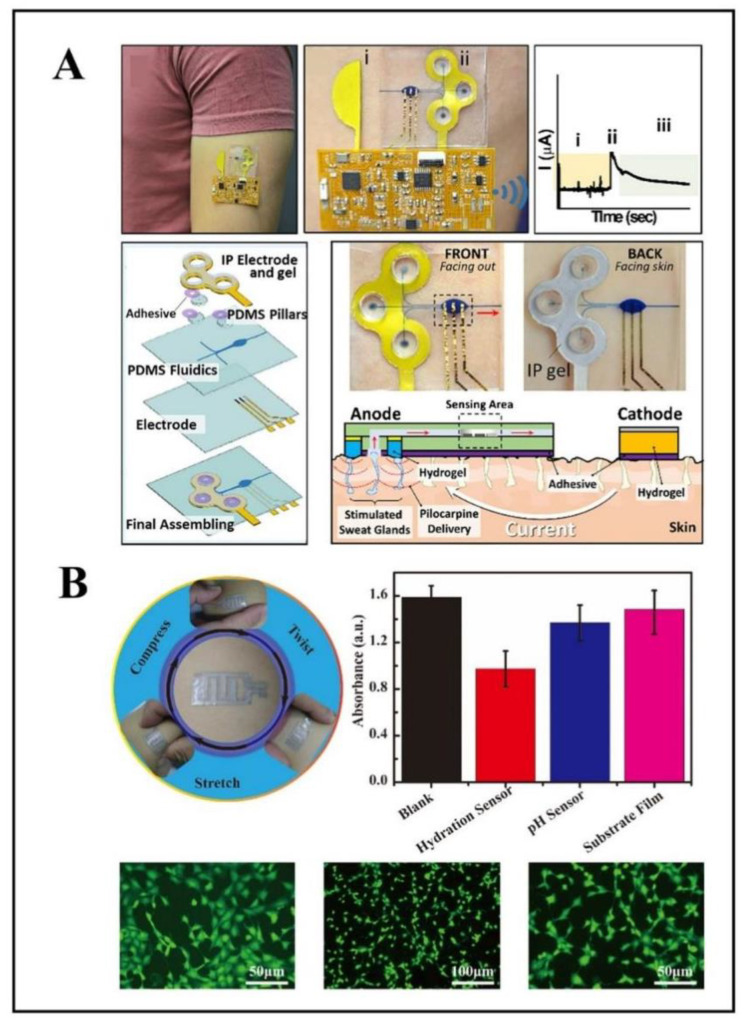
Examples of wearable flexible sensors for glucose and pH monitoring. (**A**) An all-in-one device that integrated sweat stimulation, collection, and analysis, (i) initial current when there is no sweat, (ii) the current of sweat coming into contact with the electrodes, (iii) the electrochemical response of the sensor. Reprinted with permission from ref. [96]. Copyright 2022, SpringerLink. (**B**) A non-invasive and biocompatible wearable sensor, reprinted with permission from ref. [102]. Copyright 2021, SpringerLink.

Blood glucose concentration is an essential indicator of the management of certain diseases, such as diabetes. According to the World Health Organization, 9% of adults worldwide have diabetes [90], so it is crucial to continuously monitor glucose to keep people with diabetes on track. Although routine blood glucose testing is done by sampling blood, this invasive method is uncomfortable and does not allow continuous monitoring. According to research, glucose levels in the blood correlate with biological fluids such as sweat, saliva, and tears [91,92,93]. Then, compared to the other two liquids, sweat extraction is obviously more convenient and hygienic. Therefore, sweat can be monitored by stretchable electrochemical sensors, providing important information related to blood glucose in time [91,94]. A device for the extraction and analysis of sweat was reported based on agarose hydrogels [95]. In this study, an L-lactic acid biosensor using potentiometric detection technology enabled in situ sweat extraction when the skin of the finger came into contact with the hydrogel. Recently, an all-in-one device that integrated sweat stimulation, collection, and analysis was developed using PDMS pillars and viscous tapes to construct microfluidic channels and iontophoretic electrodes near the pillar inlet area to detect glucose (Figure 6A) [96]. A sweat-stimulating drug (pilocarpine) stimulated the release of sweat, which was collected near the pillar inlets and flowed naturally into the fluid channel, finally reaching the sensing area. Glucose oxidase reacted with glucose, and the current change was measured by electrochemical monitoring technology to realize the detection of glucose. It is worth mentioning that under the action of stimulant drugs, this work does not require vigorous exercise to generate sweat, and the use of microfluidic channels avoids the tedious steps of transferring the collected sweat to the corresponding site.

### 2.6. pH

pH is another critical parameter to maintain physiological homeostasis and assess physical conditions because it is closely related to the wound-healing of human skin. Moreover, the activity of most enzymatic reactions also depends on pH, which can prompt much information about physiological conditions [97,98]. Therefore, continuous monitoring of pH also makes sense to maintain a healthy state. With the gradual popularization of pH sensor applications in various industries, especially in the field of healthcare, traditional electrodes (such as glass electrodes) can no longer meet the new needs of personalized healthcare [99]. Traditional electrodes often have shortcomings due to the lack of bending ability, difficulty in miniaturization, and brittleness during movement [100]. Flexible, wearable, low-cost, and biocompatible pH sensors have attracted considerable interest from researchers [101].

Recently, a non-invasive and biocompatible wearable sensor was presented for pH detection (Figure 6B) [102]. The sensor was formed by integrating carboxylate multiwalled carbon nanotubes into composite silk fibroin film through laser cutting and etching methods. The research is similar to electronic skin that the wearer can use for long-term health monitoring. Another flexible wearable sensor was proposed to monitor pH concentration in sweat continuously [103]. Ti_3_C_2_T_x_ (F − Ti_3_C_2_T_x_)/polyaniline (PANI) with extremely high conductivity and biocompatibility was successfully prepared and acted as the working electrode to monitor surface hydronium ions.

**Table 1:** Summary of flexible sensors for vital sign monitoring.

Biological Signals	Sensors	Flexible Materials	Features	Refs
Temperature	temperature sensor array	PANI, Ecoflex	precise response time, high resistance sensitivity	[43]
	temperature sensor array	PI, PDMS	excellent mechanical flexibility, visible transparency, self-power	[44]
	temperature sensors	PEI	ultrathin, flexible, lightweight	[46]
Blood pressure	graphene electronic tattoos	graphene	self-adhesive, low-impedance, lightweight	[55]
	piezo-composite ultrasonic sensor	PDMS	noninvasive, nonocclusive, calibration-free	[56]
ECG	ECG electrode	natural leather	convenient, comfortable, flexible, wearable	[61]
Pulse	strain sensor	PANI	flexible, low cost, wearable, simple manufacturing process	[65]
	pressure sensor arrays	PDMS, PET	flexible, wearable, multichannel	[66]
Human motion	strain sensor	conductive textile	washable, lightweight, flexible, reusable wearable	[78]
	triboelectric nanogenerator fabric sensor arrays	PDMS	flexible, stretchable, self-power	[79]
	eye-movement sensor	PDMS	highly-sensitive, skin-attachable, noninvasive	[81]
Respiratory rate	fiber Bragg gratings sensors	PI, silicone	wearable, flexible	[88]
	fiber Bragg gratings sensors	textiles	Wearable, multi-point sensing,	[89]
Glucose	sweat sensor	agarose hydrogel	noninvasive, simple, in-situ analysis	[95]
	iontophoresis integrated microfluidic epidermal biosensor	PDMS	soft, flexible, wearable, skin-mounted, non-invasive,	[96]
pH	pH sensor	composite silk fibroin film	high flexibility, biocompatibility, air permeability, biodegradability	[102]
	electrochemical pH Sensor	PET, PANI	skin-attachable, wearable, flexible	[103]

## 3. Materials

Appropriate materials, such as flexible materials, can significantly improve the performance and flexibility of wearable devices for broader applications. The difference in material properties (such as the shape, porosity, and surface area) and properties based on sensor applications (such as elastic linearity and accuracy) [104] are often important factors in distinguishing different sensors [105]. Therefore, it is necessary to research new materials suitable for flexible wearable sensors to meet the characteristics of softness, comfort, lightweight, better biocompatibility, and economical cost [10] so that the new flexible sensors can play an influential role in hospital diagnosis and home monitoring. A flexible wearable device includes three primary components: a substrate, an active element, and an electrode [1].

**Figure 7:**
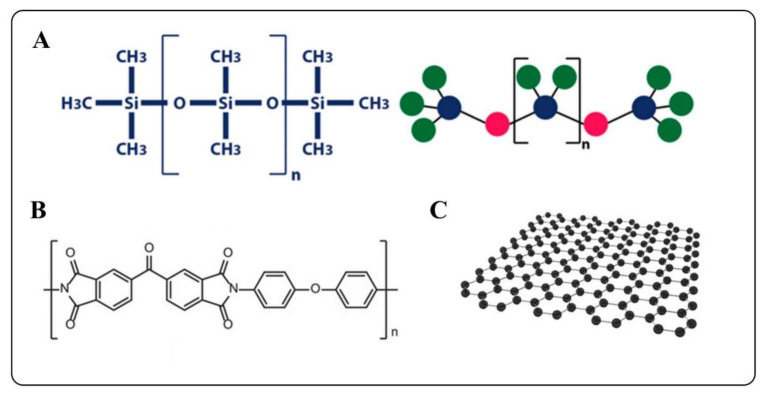
Chemical structure diagram of (**A**) PDMS, reprinted with permission from ref. [137]. Copyright 2021, MDPI, (**B**) PI, reprinted with permission from ref. [114]. Copyright 2019, Wiley, (**C**) graphene, reprinted with permission from ref. [138]. Copyright 2022, Wiley.

**Figure 8:**
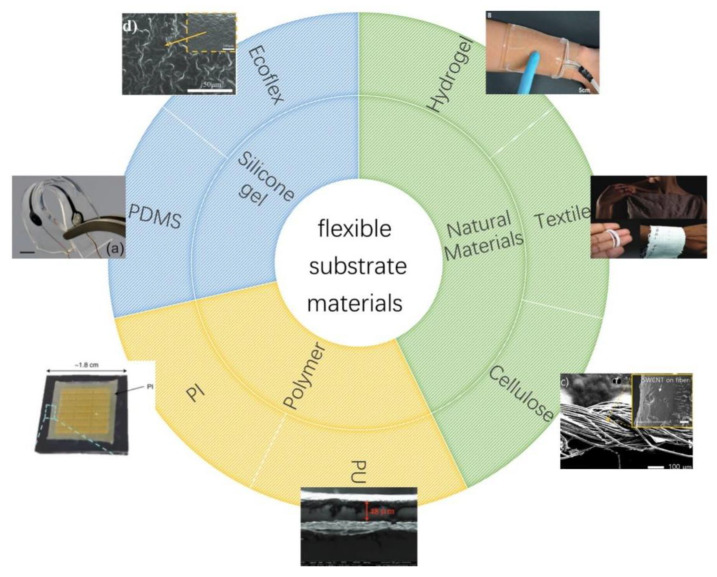
Flexible substrate materials for flexible wearable sensors, including microscopic image of Ecoflex with wavy texture structure, reprinted with permission from ref. [139]. Copyright 2021, Wiley. Integration of multi-walled carbon nanotubes and graphene nanosheets into PDMS, reprinted with permission from ref. [107]. Copyright 2013, Journal of Applied Physics. PI flexible material is covered on a Sio/Si substrate, reprinted with permission from ref. [140]. Copyright 2021, nature. Cross-sectional microscopic image of PU, reprinted with permission from ref. [141]. Copyright 2015, nature. Single-wall carbon nanotube-coated cotton thread, reprinted with permission from ref. [142]. Copyright 2018, American Chemical Society. Textile with LED embedded yarns, reprinted with permission from ref. [143]. Copyright 2018, Nature. Touch panel with hydrogel as the flexible substrate, reprinted with permission from ref. [144]. Copyright 2016, Science.

For flexible wearable sensors, it is essential to use a flexible substrate to impart stability to the active material, so the flexible substrate material is also the key to determining the comfort of the sensor. In this regard, metal foils and organics like flexible polymers, silicones, and rubbers are commonly used flexible substrates [105]. Since most wearable sensors are in direct contact with human skin, using suitable biocompatible materials as substrates tends to ground many hazards [106]. PDMS are very commonly used flexible substrates, which are commercial silicone elastomers with good biocompatibility, non-toxic and non-oxidative properties, high flexibility, and high thermal expansion coefficient [107]. The chemical structure of PDMS is shown in Figure 7A. However, the inherent mechanical properties of PDMS are poor, which also limits its application in fields that require strong mechanical properties. In order to overcome this obvious shortcoming, bulk modification methods are used to change the chemical structure of PDMS, and then surface functionalization is used to meet the special needs of various fields, such as strong adhesion [108]. Therefore, modified PDMS is considered to be a crucial flexible substrate in artificial intelligence and medical fields. In addition, silicone rubber, such as Ecoflex, has also been used in sensors with high performance [109,110]. Among the polymer substrates, PI membranes exhibit extreme flexibility and can work under high temperatures and strong acid–base conditions [111], making PI and micromechanical systems fabrication process compatible [112,113]. Therefore, PI is considered one of the essential polymer dielectrics [114]. To date, various types of PIs with different functions have been synthesized for flexible substrates. The chemical structure of polyimides is shown in Figure 7B. Polyurethane (PU) is often used to manufacture sensors, such as temperature sensors, because of its good stretchability and economic efficiency [115,116]. In order to avoid environmental pollution caused by large-scale use, researchers continue to explore degradable materials, such as natural materials. Cellulose paper is cheap, flexible, inherently hydrophilic, and degradable [117,118]. Therefore, the research and use of materials based on cellulose paper have effectively promoted the development of flexible wearable sensors [119]. At present, sensors based on cellulose paper emerge in an endless stream, such as strain sensors [106], biophysical sensors [120], self-powered sensors [121], transistors [122], and RF antennas [123]. Strain sensors made from elastic cellulose can be sewn into clothing and integrated with wire yarns to create flexible wearable sensors [124]. Natural textile materials can be integrated with conventional rigid sensors, which are also ideal for flexible materials [97]. Flexible, stretchable, and biocompatible hydrogels have also been used in a variety of flexible wearable sensors. The powerful functional properties make hydrogels one candidate for replacing traditional sensors [125]. A summary of common flexible substrate materials is shown in Figure 8.

Conductive electrodes and active elements are also critical components and can generally be embedded on top of flexible substrates. The development of medical sensors strongly depends on the advances in nanomaterials [126,127]. The outstanding flexibility and miniaturization of flexible polymers and nanomaterials play an important role in sensor construction and have been used in developing of high-performance sensors [128]. Thin, flexible sensors made of PVDF polymers find wide application in healthcare monitoring [1]. Graphene possesses excellent physical and chemical properties, such as high carrier mobility, high thermal conductivity, excellent mechanical properties, and light transmittance [129,130,131]. The structure of graphene is shown in Figure 7C. In addition, the transparency and adhesion brought by the ultra-thin thickness of graphene also have outstanding application potential in flexible electrodes [132]. Therefore, it has been widely used in medical sensors, including artificial skin [133], artificial throat [134], and wearable human–machine interfaces [135]. More significantly, in order to use energy efficiently, graphene materials are often used as electrode materials to meet the demand for new energy storage energy [136]. Graphene is the most promising active material for developing flexible wearable devices.

## 4. Sensor Fabrication Techniques

In order to produce more efficient and inexpensive, flexible wearable sensors, manufacturing technology should have simple operating steps and inexpensive equipment. Fabrication of conventional wearable sensors often requires standard photolithographic processes achieved by using light to pattern a design on a substrate [145]. However, the limitations of processable materials and film thicknesses make them unsuitable for flexible sensors [1]. In order to realize the fabrication of wearable devices from flexible substrates, new fabrication methods need to be explored.

**Figure 9:**
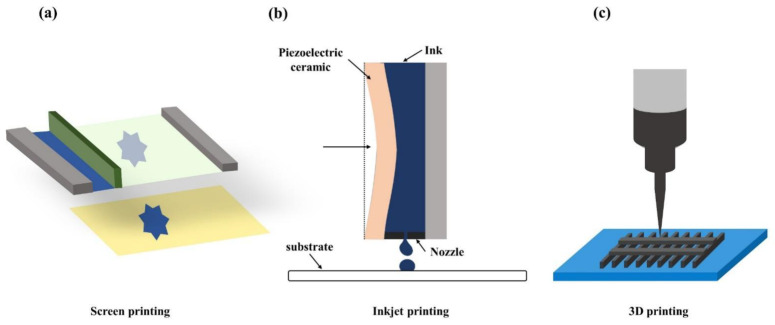
A visual summary of printing technology. (**a**) Screen printing: the ink is transferred to the sublayer with the mask. (**b**) Inkjet printing: deformation extrusion is generated by piezoelectric crystals to squeeze the ink in the nozzle. (**c**) 3D printing: the material is printed out of a three-dimensional object by a printing device.

There are a variety of methods for processing flexible substrates for various types of sensors. Among them, functional printing technology seems to have the most potential and cost-effective approaches [146], including inkjet printing, screen printing [147], and 3D printing [94], because of its high utilization of raw materials, simple processing technique, and low cost [148]. A visual summary of printing technology is shown in Figure 9.

Screen printing technology, transferring a paste-like material (ink) onto a substrate with masks, is mainly used to produce working electrodes of electrochemical and electromechanical sensors [147]. The most significant advantage of using this technique is the versatility in the type of materials that can be used as a substrate for the ink [149]. A flexible sensor focusing on wound monitoring was presented with high specificity and reliability based on the flexible paper substrate [150]. Electrochemical-based sensor working electrodes were fabricated on flexible paper substrates using a simple, low-cost screen-printing technique. Inkjet printing refers to the inkjet printing of ink materials onto flexible substrates to form specific structures [151]. This technique pushes functional inks to print accurately and quickly and is developed from the laser printers used for printing documents [149]. Recently, inkjet printing was applied to construct a wearable sensor behind the ear for monitoring EEG signals [152]. The working electrode of the sensor was printed on the tattoo sticker and connected to the skin through the adhesive separation layer due to its lightness and flexibility. 3D printing is the best way to develop exotic features [1]. For example, a wearable flexible sensor with high toughness and self-processing hydrogen was created using light energy 3D printing technology for fabrication [153]. A high-throughput microfluidic sweat-sensing patch was developed using R2R rotary screen printing, which is high-throughput [154]. However, the pattern resolution of printing technology was not yet sufficient for complex geometries. Therefore, a series of high-performance stretchable devices, combining printing and lithography technology, was demonstrated to achieve a breakthrough [155].

## 5. Power Supply

Another breakthrough in flexible wearable sensors lies in the innovation of power supply methods. Power devices are essential components of wearable systems [8]. Powering the sensor over a wired connection has a number of drawbacks. On the one hand, it cannot meet the small size of flexible wearable devices. On the other hand, the sensor device cannot be used during charging, which cannot meet the original intention of medical monitoring continuity [94]. Therefore, the power supply is the key to achieving continuous monitoring [156,157]. Real-time applications for monitoring different signals depend on wireless power. Therefore, it is vital to explore a new power supply method.

Currently, many wireless power sources have been applied to wearable devices, among which near-field communication (NFC) technology is undoubtedly the most eye-catching [158]. NFC is not only used for data exchange between various devices but also to power wearable devices. A versatile wearable device was proposed for monitoring ECG, body temperature, and sweat, with NFC to power the device [159]. However, NFC still has limitations, as it can only be used for monitoring devices with low energy consumption, and there are certain requirements for distance. In addition, emerging autonomous sensors and devices can also be powered using ambient energy sources, such as mechanical energy from human movement or energy from thermal changes and light [160]. For example, an electronic skin based on a flexible thermoelectric conversion has been reported, which can harvest energy from the body and provide a continuous energy supply [161]. Recently, another self-powered electronic skin that combined the triboelectric effect with thermoelectric conversion has been proposed, which can simultaneously convert the monitored temperature and pressure into independent signals [162]. Such self-powered sensors often do not require the integration of additional power modules, which provides a new solution for miniaturized flexible sensors. Triboelectric sensors using PVDF-TrFE, BaTiO3 nanoparticles materials, and magnet-based magnetic sensors have achieved promising results [163]. For example, a self-powered sensor capable of monitoring complex signals in human physiology was designed, which used magnetron sputtering technology to coat ZnO nanomaterials onto the surface of PVDF nanofibers with excellent flexibility [15]. The three-dimensional structure formed by the overlapping extension of PVDF/ZnO composites has excellent electrochemical performance and good biocompatibility and gas permeability. Another triboelectric sensor with a multilayer structure provides a new platform for flexible medical monitoring [164]. In this work, PVDF-TrFE and BaTiO3 nanoparticles were formed into the multilayer composite structure, and mechanical energy was converted into electrical energy using triboelectric and electrostatic induction principles. The sensor possessed high flexibility and charge density by combining the two materials. Similarly, a successful design for monitoring physiological signals was a self-powered sensor based on magnetoelastic effects [165]. Electricity was generated by the change in the magnetic field of the miniature magnet covering the ultra-thin and soft silicone polymer. In addition to providing sustainable power, the sensor also acts as a heart rate monitor and is waterproof, ensuring data accuracy even when sweating.

## 6. Conclusions

Different from traditional rigid sensors, flexible wearable sensors have unique advantages in terms of comfort and flexibility and can be in direct contact with human skin and tissues. Therefore, it is suitable for the detection of various vital signs. Although a lot of work has been done on flexible wearable sensors, there are still some challenges. An important feature of flexible wearables is continuity. Based on this feature, a large amount of data will be generated. Consequently, this may lead to the private data leakage of users, resulting in security risks. In addition, it will also be essential to filter out important data from a large amount of data. Wearable sensors are often designed to be as small and compact as possible to fit the skin. However, most materials applied to wearable sensors may not be safe and reliable, increasing the risk of wound deterioration. Therefore, it is very important to choose the appropriate manufacturing process and flexible materials to avoid environmental pollution and harm to users. If soft and self-healing materials can be used as the substrate, it will significantly improve the service life of the sensor. It is worth noting that the batteries of most flexible sensors cannot meet the demand for long-term power supply. At present, there are many types of wearable sensors on the market, and people’s monitoring needs are gradually increasing with the increase of sensor types. In order to meet the needs of users, multi-functional sensors will be the future development direction. On this basis, smart sensors that are selective enough to meet monitoring needs will also attract much attention. However, poor sensitivity and stability, complex operating procedures, and expensive manufacturing equipment hinder the pace of large-scale industrial production. Finally, the crosstalk of sensor signals and the effects of harsh environments can interfere with analytical data and affect the stability of the sensor system. Improving the sensitivity and selectivity of the sensor is a very effective solution. In addition, the circuit is designed to be cleaner to avoid potential noise. Overall, despite the challenges in developing flexible wearable sensors for medical monitoring, many positive signs remain encouraging.

## Data Availability

Not applicable.
